# OCT4 and NANOG are involved in adrenocortical tumorigenesis

**DOI:** 10.20945/2359-4292-2026-0055

**Published:** 2026-05-01

**Authors:** Marcelo M. Cavalcanti, Mariana P. Halah, Letícia F. Leal, Fernanda B. Coeli-Lacchini, Carlos A. Scrideli, Carlos A. F. Molina, Jose A. Yunes, Silvia Brandalise, Ayrton C. Moreira, Fernando Ramalho, Leandra N. Ramalho, Margaret de Castro, Sonir R. Antonini

**Affiliations:** 1 Departamento de Pediatria, Faculdade de Medicina de Ribeirão Preto, Universidade de São Paulo, Ribeirão Preto, SP, Brasil; 2 Hemocentro de Ribeirão Preto, Faculdade de Medicina de Ribeirão Preto, Universidade de São Paulo, Ribeirão Preto, SP, Brasil; 3 Departamento de Cirurgia, Faculdade de Medicina de Ribeirão Preto, Universidade de São Paulo, Ribeirão Preto, SP, Brasil; 4 Centro Infantil Boldrini, Universidade Estadual de Campinas, Campinas, SP, Brasil; 5 Departamento de Clínica Médica, Faculdade de Medicina de Ribeirão Preto, Universidade de São Paulo, Ribeirão Preto, SP, Brasil; 6 Departamento de Patologia, Faculdade de Medicina de Ribeirão Preto, Universidade de São Paulo, Ribeirão Preto, SP, Brasil

**Keywords:** Adrenal cortex neoplasms, pathogenesis, adrenocortical carcinoma, pediatric

## Abstract

**Objective:**

To investigate the involvement of regulators of pluripotency and self-renewal of embryonic stem cells in adrenocortical tumors (ACT).

**Subjects and methods:**

Clinicopathological data and tissues from 114 ACT patients (96 pediatric-pACT; 18 adults) were analyzed. Normal fetal (n = 33) and postnatal adrenals (n = 26) were used as controls. STAT3, NANOG, SOX2, and OCT4 expression was evaluated by qPCR and IHC. We evaluated intracellular NANOG localization by immunofluorescence and its interaction with beta-catenin after inhibiting the Wnt pathway in a beta-catenin-mutated ACT cell line (NCI-H295).

**Results:**

IHC showed NANOG, OCT4, SOX2, and STAT3 expression in fetal adrenals until mid-pregnancy, disappearing thereafter. Positive OCT4 nuclear staining was found in 32% of pACT samples and was associated with metastasis (OR = 2.28; 95% CI:1.13-4.59; P < 0.05). ACTs presented lower SOX2 mRNA expression (P < 0.01). STAT3 mRNA levels were higher in cortisol-secreting ACT (P = 0.01) and in adult adenomas (P < 0.01). NANOG mRNA was higher in p.S45P CTNNB1 mutated ACT (P < 0.01). NCI-H295 cells exhibit nuclear NANOG expression, which was decreased by inhibiting the Wnt/beta-catenin pathway (P < 0.01).

**Conclusion:**

Markers of pluripotency and self-renewal of embryonic stem cells are expressed until mid-pregnancy, contributing to the adult adrenal stem cell niche. They are absent postnatally but are expressed in a subset of ACT. Specifically, pS45P beta-catenin-mutated ACTs express more NANOG. Increased OCT4 expression in pACT is associated with worse prognosis, and inhibiting the Wnt/beta-catenin pathway in these cells impairs NANOG expression.

## INTRODUCTION

Deregulation of important signaling pathways that play significant roles in orchestrating early embryonic development and morphogenesis has been previously described in adrenocortical tumorigenesis ^(1-5)^. They include wingless-type (Wnt), Hedgehog (HH), and Notch, all of which are involved in the balance between stem cell self-renewal and differentiation ^(6,7)^.

The canonical Wnt/β-catenin pathway is involved in stem cell proliferation, differentiation, and migration within their niche ^(8,9)^. Activation of the Wnt pathway has been consistently observed in adrenocortical tumorigenesis, either through activating mutations in *CTNNB1* in pediatric ^(1)^ and adult adrenocortical tumors (ACT) ^(5)^, or through *ZNRF3* mutations and deletions in adult tumors ^(10,11)^. Additionally, our previous data have shown that abnormal beta-catenin accumulation was present in most pediatric ACTs (pACT), even those without *CTNNB1* mutations, suggesting other mechanisms might be involved ^(1)^.

HH signaling regulates stem cell proliferation and differentiation in the central nervous system ^(7)^, skin ^(7)^, intestine ^(12)^, mammary gland ^(13)^, pancreas ^(14)^, and adrenal cortex ^(4)^. In adrenocortical tumorigenesis, we previously showed that adult adrenocortical carcinomas (ACC) exhibit higher expression of Sonic Hedgehog (SHH) pathway components, including *PTCH1*, *SMO*, *GLI3*, and *SUFU*, compared with normal adult adrenal cortices ^(4)^. Conversely, pACT showed lower mRNA expression of *SHH*, *PTCH1*, *SMO*, *GLI1*, and *GLI3* than control pediatric adrenal cortices ^(4)^.

Notch signaling acts through intercellular interactions in several tissues, maintaining stem cell proliferation and determining stem cell fate ^(15)^. The activation of Notch signaling through the upregulation of *JAG1* has been reported in a variety of cancers, including ACC ^(2,16)^. Moreover, in ACC, *JAG1* seems to be overexpressed in a subgroup of patients with better clinical outcomes ^(17)^.

Octamer-binding transcription factor 4 (OCT4), signal transducer and activator of transcription 3 (STAT3), SRY-box transcription factor 2 (SOX2), and NANOG are key transcription factors implicated in cancer biology ^(18-21)^. They are master regulators of pluripotency and self-renewal in embryonic stem cells, either by interacting with each other or by regulating pathways involved in stem cell proliferation, such as the LIF/STAT3 pathway ^(18-21)^. They have central roles in maintaining cancer stem cell (CSC) properties and promoting aggressive tumor behavior. In the adrenals, NANOG, OCT4, and STAT3 are expressed only during early embryonic stages in murine embryos (prior to E9), before steroidogenic function begins, suggesting that these proteins maintain adrenal cortex cells in an undifferentiated state ^(22-25)^. There is little data on the expression pattern of NANOG, OCT4, SOX2, and STAT3 during human adrenal development.

Together or separately, increased NANOG, OCT4, and SOX2 expression has been associated with tumorigenesis, metastasis, and/or recurrence in poorly differentiated breast cancer, glioblastomas, bladder carcinomas, squamous cell carcinoma of the skin, prostate cancer, and head and neck squamous carcinoma ^(26-29)^. One previous study reported that STAT3 positive staining was more frequent in ACC than in adrenocortical adenomas ACA ^(30)^. There is likely a link between the mechanisms that maintain stem cells and the abnormal molecular pathways involved in adrenocortical tumorigenesis. Nevertheless, the potential involvement of the stem cell markers NANOG, OCT4, SOX2, and STAT3 in ACT has not been thoroughly investigated in these tumors.

OCT4 is aberrantly expressed in numerous malignancies and is enriched in CSC populations, regulating self-renewal, pluripotency, epithelial–mesenchymal transition, and therapy resistance ^(31,32)^. In addition to its cell-intrinsic effects, OCT4 contributes to tumor progression by shaping the tumor microenvironment through enhanced angiogenesis, immune evasion, and metabolic reprogramming ^(31,32)^. Co-expression of OCT4 and Nanog has been linked to more aggressive clinicopathological features and poorer outcomes in some cancers ^(33,34)^.

STAT3 is essential for CSC maintenance and is activated in many cancer types, acting as a major oncogenic driver by regulating proliferation, survival, angiogenesis, immune suppression, and resistance to therapy ^(35)^. Its activation correlates with an unfavorable prognosis, and it cooperates with other stemness-associated transcription factors, including Nanog and SOX2, to sustain CSC properties ^(35-37)^.

SOX2 is frequently overexpressed across solid tumors and supports CSC maintenance and is associated with poor prognosis, lymph node metastasis, and disease recurrence ^(38)^. It interacts with multiple oncogenic signaling pathways, including STAT3 and Wnt/β-catenin, to drive tumor progression and cellular plasticity ^(38)^.

NANOG is a master regulator of pluripotency and self-renewal in CSCs ^(33,34)^. High NANOG expression is linked to advanced tumor stage, metastatic spread, chemo resistance, and poor clinical outcomes in a wide range of malignancies ^(39)^. Nanog promotes tumorigenesis through activation of pathways such as JAK/STAT, Wnt/β-catenin, and TGF-β, and its co-expression with OCT4 further strengthens CSC traits ^(39)^.

Together, these transcription factors orchestrate the stemness, plasticity, and aggressive phenotype of cancer cells. By sustaining CSC survival and therapy resistance, they represent promising targets for therapeutic strategies to eliminate CSCs and improve long-term treatment outcomes. There is limited research directly utilizing the markers OCT4, STAT3, SOX2, and NANOG to study adrenocortical tumors.

Therefore, we performed a study to assess the expression of these markers in ACT. First, we evaluated the expression patterns of NANOG, OCT4, SOX2, and STAT3 in normal adrenal cortex across different developmental stages, including fetal and postnatal adrenal cortices. Then, we evaluated mRNA and protein expression of *NANOG*, *OCT4*, *SOX2*, and *STAT3* genes in pediatric and adult ACTs and their association with clinical features. In addition, we analyzed the in vitro interaction between Wnt/β-catenin signaling and *NANOG*.

## MATERIALS AND METHODS

### Experimental design

**Figure 1.**
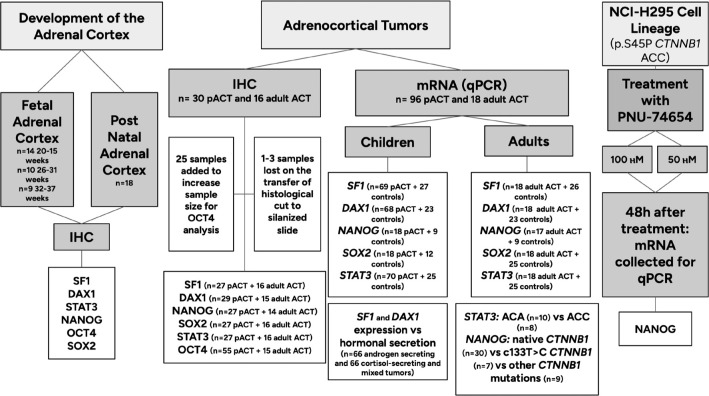
Experimental design.

IHC: immunohistochemistry; qPCR: quantitative polymerase chain reaction; pACT: pediatric adrenocortical tumors; ACT: adrenocortical tumors, ACA: adrenocortical adenoma; ACC: adrenocortical carcinoma.

Experimental design is detailed in **Figure 1**. Different sample sizes were used for each experiment due to limitations on the number of samples and the quantity of material available. Protein expression of SF1, DAX1, NANOG, OCT4, SOX2 and STAT3 was evaluated. Due to the availability of biological material, it was performed in a subset of pACT samples. In each analysis, in the results section, the exact number of samples is specified.

### Controls – Fetal and postnatal normal adrenal cortices

For the immunohistochemistry (IHC) study, we evaluated a group of 33 normal prenatal adrenal paraffin-embedded tissues obtained from spontaneously miscarried fetuses grouped according to gestation age: 20–25 weeks (n = 14), 26–31 weeks (n = 10), 32–37 weeks (n = 9). As postnatal controls, we evaluated 18 adrenals from children between birth and 10 years old who underwent routine autopsy examination in our Department of Pathology; fetuses or children with multiple malformations or children with known endocrine diseases, or previous use of glucocorticoids were excluded. Also, 26 specimens of normal adrenal cortices were analyzed as controls in quantitative polymerase chain reaction (qPCR) experiments: 12 pediatric specimens obtained after surgical treatment of Wilms’ tumor and 14 from adult subjects with sudden death who underwent routine autopsy.

The surgical tissues were collected at the time of surgery. The autopsy specimens and the fetal tissues from miscarriages were collected at the moment of the evaluation by the Pathology Department, which occurred a couple of hours after death or miscarriage or at a maximum period of 12 hours after the patient’s death or the occurrence of miscarriage if the event was during the night shift to minimize degradation and variability in both IHC and PCR analyses.

Information regarding the origin of each pediatric control and fetal tissue is on **Supplementary Table 1**, and the adult control tissue information is on **Supplementary Table 2**. Fetal tissues originated from spontaneous miscarriages; autopsy-derived tissues were obtained from individuals without clinical or pathological evidence of endocrine or neoplastic disease processes, and who did not use glucocorticoids; surgical control specimens were collected from patients undergoing procedures for non-inflammatory, non-neoplastic indications.

### Patients

We evaluated 114 patients with ACT (96 Pediatric and 18 adults) who were diagnosed and followed at two reference centers in southeastern Brazil (**Tables 1 and 2**). According to the experiment, the entire cohort or a subset of it was analyzed. Routine diagnostic tests and imaging evaluations were performed before surgical treatment, and the patients were followed up after surgery, as previously reported ^(1,4,5)^. Tumor stages were classified according to the International Pediatric Adrenocortical Tumor Registry for children and Macfarlane modified by Sullivan ^(40)^ for adults. In adult ACT, a Weiss score of less than three was used to classify the tumors as ACA, and a score of three or greater was used to classify them as ACC ^(41)^. In the pediatric group, we also assessed the Weiss score but did not categorize ACT as ACA or ACC according to this criterion because it is not entirely applicable and does not accurately predict clinical outcomes in this age group ^(41)^. This study was approved by the Ethics Committee (HCRP Process 3019/2013). Informed written consent was obtained from patients and controls or their parents.

**Table 1.** Clinicopathological data of pACTs

**Gender**		Male		18	19%
		Female		78	81%
**Deaths**		Alive		74	77%
		Dead		15	16%
		Unknown due to loss of follow up		7	7%
**Age at diagnosis (years)**		Median		1.6	
		Minimum; maximum		0.4; 15.6	
**Weiss score**		0		2	2.1%
		1		3	3.1%
		2		1	1.0%
		3		4	4.2%
		4		15	15.6%
		5		10	10.4%
		6		25	26.0%
		7		15	15.6%
		8		11	11.5%
		9		8	8.3%
		Unknown		2	2.1%
		Deaths	>5 year survival		
**Tumor Stage**	I	4 (6.7%)	35 (58.3%)	60	62.5%
	II	3 (21.4%)	9 (64.3%)	14	14.6%
	III	1 (7.7%)	5 (38.5%)	13	13.5%
	IV	7 (77.8%)	2 (22.2%)	9	9.4%
**Relapse and/or Metastasis**		Yes		18	18.8%
		No		52	54.2%
		Unknown		26	27.1%
**Chemotherapy**	Yes			19	19.8%
	No			48	50.0%
	Unknown			29	30.2%
**Surgery**	Yes			96	100%
		Female	Male		
**Hormone Secretion**	Virilizing	43	8	51	53.1%
	Mixed (Virilizing + Cushing)*	32	9	41	42.7%
	Non secreting	3	1	4	4.2%

* Clinical or subclinical Cushing syndrome.

**Table 2.** Clinicopathological data of adult ACTs

**Gender**		Male		2	11%
		Female		16	89%
**Deaths**		Alive		14	78%
		Dead		3	16%
		Unknown due to loss of follow up		1	6%
**Age at diagnosis (years)**		Median		42.5	
		Minimum; Maximum		21; 66	
**Weiss score**		0		1	5.5%
		1		7	38.9%
		2		2	11.1%
		3		2	11.1%
		4		3	16.7%
		5		0	0%
		6		3	16.7%
		Deaths	>5 year survival		
**Tumor Stage**	I	0	3 (42.8%)	7	38.9%
	II	1 (16.7%)	4 (66.7%)	6	33.3%
	III	0	0	1	5.6%
	IV	2 (50%)	1 (25%)	4	22.2%
**Relapse and/or Metastasis**		Yes		5	2.,8%
		No		13	72.2%
**Chemotherapy**	Yes			6	33.3%
	No			12	66.7%
**Surgery**	Yes			18	100%
		Female	Male		
**Hormone Secretion**	Virilizing	2	0	2	11.1%
	Cushing	7	0	7	38.9%
	Mixed (Virilizing + Cushing*)	5	2	7	38.9%
	Non functioning	2	0	2	11.1%

* Clinical or subclinical Cushing syndrome.

### Sample size

Owing to the rarity of the condition, we employed a convenience sampling approach for the pediatric and the adult cohort. We included all consecutive eligible patients identified during the study period. This sample size reflects the maximum feasible recruitment for this population in our University Hospital during the study period. For each experiment, either qPCR or IHC, the number of samples of pACT, adult tumors and controls is specified in the results and was selected according to tissue availability.

### IHC analyses

#### Standardization of IHC analyses

**Supplementary Figure 1.**
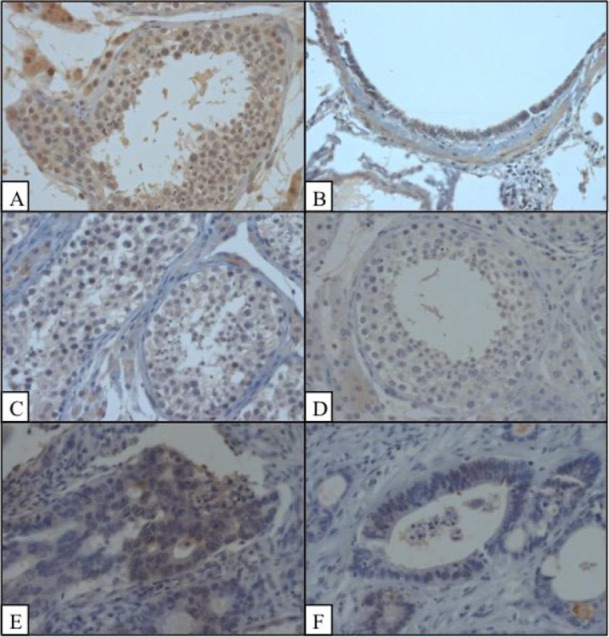
IHC photos of positive controls used to standardize the antibodies. Testicular tissue marked with anti-SF1 (**A**), anti-OCT4 (**C**), and anti-NANOG (**D**). Lung tissue marked with anti-DAX1 (**B**). Colon tissue marked with anti-SOX2 (**E**) and anti-STAT3 (**F**).

Paraffin blocks containing tissues known to express the target proteins (identified from manufacturer information or the Human Protein Atlas) were sectioned at 5 µm onto poly-L-lysine–coated slides. Sections were deparaffinized overnight, cleared in xylene, and rehydrated through graded alcohols. Heat-induced antigen retrieval was performed in Diva Decloaker 10X^®^ buffer (Biomedical Care, CA, USA) at 90 °C for 40 min, followed by cooling in distilled water. IHC staining was carried out using the REVEAL Polyvalent HRP-DAB Detection System^®^ (Spring Bioscience, CA, USA). Endogenous peroxidase was blocked for 15 min, followed by protein blocking for 10 min. Primary antibodies were tested in serial dilutions (1:25–1:300) and incubated overnight at 4 °C. After washing, slides were incubated with the rabbit anti-mouse complement for 10 min, HRP conjugate for 15 min, and DAB for 5 min. Hematoxylin counterstaining was performed for 15 s, followed by bluing, dehydration, clearing, and mounting. Optimal antibody dilutions were determined from the positive control slides (**Table 3**), with representative images shown in **Supplementary Figure 1**.

**Table 3.** List of antibodies used in immunohistochemistry experiments, tissues used as positive controls, and dilutions

Antibodies (Manufacturer)	Positive-Control	Dilution
Anti human SF-1 mouse monoclonal antibody PP-N1665-00 (Perseus Proteomics^®)^	Testicle	1:25
Anti-NR0B1/DAX1 antibody ab60144 (Abcam^®)^	Lung	1:25
Anti-STAT3 (phospho Y705) antibody [EP2147Y] ab76315 (Abcam^®)^	Colon	1:25
Anti-SOX-2 (Y-17) sc-17320 (Santa Cruz Biotechnology^®)^	Colon	1:25
Anti-NANOG antibody - ChIP Grade ab21624 (Abcam^®)^	Testicle	1:25
Anti-OCT-3/4 (C-10) sc-5279 (Santa Cruz Biotechnology^®)^	Testicle	1:50

#### Fetal and postnatal adrenal cortices

The adrenal morphology was evaluated by routine hematoxylin-eosin staining. We performed the IHC studies using the primary antibodies described previously (**Table 3**). Labeling was developed with 3,3-diaminobenzidine (DAB; Vector Laboratories Inc.), and the nuclei were stained with Harris hematoxylin. For the analyses, we subcategorized our samples into gestational and postnatal age groups: 20–25 weeks (n = 14), 26–31 weeks (n = 10), 32–37 weeks (n = 9), and postnatal (n = 18). These samples were analyzed by M.M.C. and confirmed by an experienced pathologist (L.Z.R.). We present a descriptive analysis of each developmental stage.

#### Tumors

Depending on the availability of paraffin-embedded samples, IHC studies to analyze STAT3, NANOG, SOX2, and OCT4 proteins were initially performed in a subset of 46 ACT samples (30 pediatric and 16 adult samples) from the University Hospital of the Ribeirao Preto Medical School. During the transfer of the histological cut to the silanized slide, some samples were lost. Therefore, the number of samples differs between analyses. In each analysis, in the results section, the exact number of samples is specified.

Preliminary analysis of our group’s data showed that increased OCT4 expression was associated with recurrence and/or metastasis. Therefore, we decided to improve our sample size and added 25 pACT samples from Centro Infantil Boldrini in the analysis. The use of these samples was approved by this collaborative center’s ethic committee (07/12). As negative controls, all specimens were incubated with an isotope-matched control antibody under identical conditions. Preparations for each marker were evaluated randomly in at least ten representative high-power fields (x400) by an experienced pathologist (L.N.R.). Two samples stained for STAT3 and three stained for NANOG had to be excluded due to the bending of the material during preparation.

The nuclear staining of the tumor samples was evaluated using the H score. The intensity score of this staining was classified on a scale ranging from 0 (absent) to 3 (strong). In addition, the percentage of positive staining tumor cells observed in each sample was categorized into a positivity score as follows: 0% - score 0; 1 to 9%, score 0.1; 10-49%, score 0.5, and ≥ 50% score 1.0. Then, we multiplied this positivity score (0, 0.1, 0.5, or 1) by the intensity score (0, 1, 2, or 3) to obtain a final semi-quantitative score (H score), ranging from 0 to 3. Although a formal inter-observer reliability analysis was not conducted, all immunohistochemical evaluations were performed by the pathology team of our University Hospital and reviewed by a pathologist who is an expert in adrenal diseases (L.Z.R.) and the first author of this study (M.M.C.). This process helped ensure a shared interpretive framework.

### RNA extraction, DNAse digestion, and cDNA synthesis

Total RNA was extracted using Trizol Reagent (Invitrogen^®^, Karlsruhe, Germany) according to the manufacturer’s protocol. RNA integrity was checked according to the 28S/18S rate, with an acceptable range of 1.6 to 2.0, and confirmed by 1.2% agarose gel. RNA concentrations were quantified by spectrometry (Nanodrop2000, Thermo Fisher Scientific Inc., Waltham, MA, USA).

To guarantee a pure RNA without DNA contamination, RNA samples (1 µg) were exposed to DNase (1 µL) for 15 min at room temperature, and then it was inactivated with 1 µL EDTA 25mM for 10 min at 65°C. mRNA was reverse-transcribed using MultiScribe™ enzyme and the High-Capacity cDNA Reverse Transcription Kit (Life Technologies). cDNA was diluted (1:5) for qPCR.

### mRNA Quantification by qPCR

The mRNA expression levels were measured in duplicate using TaqMan assay (Life Technologies, Foster City, CA, USA) according to the manufacturer’s recommendation. The target genes evaluated were *STAT3* (Hs01047580_m1), *NANOG* (Hs04260366_g1), *SOX2* (Hs01053049_s1), and *OCT4* (Hs01654807_s1). The 2-ΔΔCt method was used to determine the relative expression of mRNA. The results were normalized with *GUSB* (Hs99999908_m1) as endogenous control. In the present study, *GUSB* was chosen as the reference gene because it follows the same standardization that was previously published by our group in gene expression analyses in adrenocortical tumors ^(4)^. In this previous study, we evaluated three extensively used endogenous controls (*TBP*, *GUSB* e *GAPDH*), and we observed that *GUSB* presented with the most stable expression in all adrenocortical human tissues. Considering this previously stablished and published validation, we have chosen *GUSB* and an endogenous control, keeping methodological consistency with the work already published by our group. The median obtained from the 2-ΔΔCt adrenal tumor tissue value was compared with the median value obtained from 2-ΔΔCt of normal adrenal tissue, obtaining the value which shows the number of times (fold) that this gene is hyper- or hypo-expressed in tumor tissue compared to control tissue ^(42)^.

### Immunofluorescence (IF)

Cells were cultured on coverslips placed in 24-well plates for the immunofluorescence (IF) assays. The NCI-H295 medium was collected, and cells were fixed in methanol for 3 minutes, washed 3 times with 0.01 M PBS, and incubated with 10% normal horse serum for one hour for blocking. Sections were then incubated overnight at room temperature with anti-NANOG primary antibody (dilution: 1:100, #610154, BD Biosciences). Next, cells were washed 3 times with 0.01 M PBS, incubated with secondary antibody CyTM5-conjugated AffiniPure donkey anti-mouse antibody (Jackson Immuno Research, #715-175-150, dilution: 1:250; red color) for four hours, and washed 3 times with 0.01 M PBS. For nuclear counterstaining, cells were incubated with 4’, 6-diamidino-2-phenylindole (DAPi; Cell Signaling Technology, #4083, dilution: 1:25,000) for 2 minutes, washed in 1X PBS, and slides were set with Fluoromount (Sigma-Aldrich). Expression and localization of NANOG were observed with a Leica TCS SP5 laser scanning confocal microscope (Leica Microsystems, Wetzlar, Germany) with fixed exposure time for all samples.

### PNU-74654 treatment of H295 cells

The NCI-H295 adrenocortical cell line was kindly provided by Professor Claudimara Lofti (University of Sao Paulo) and was cultured in RPMI 1640 medium (GIBCO, Life Technologies, Foster City, CA) supplemented with 2% fetal bovine serum (Sigma Aldrich, St. Louis, MO, USA), 1% ITS Premix (BD Biosciences) and 1% penicillin/streptomycin (100 mg/mL; GIBCO Life Technologies) and harvested weekly. Cells were cultured in a monolayer and maintained under standard conditions at 37°C in a humidified atmosphere (5% CO2 – 95% air). The NCI-H295 carries the p.S45P CTNNB1 mutation, which constitutively activates the Wnt pathway signaling.

To assess the potential interaction between the Wnt/β-catenin pathway and *NANOG* mRNA expression, we analyzed *NANOG* mRNA levels in NCI-H295 cells under basal conditions and after the treatment of these cells with different doses of PNU-7654 over standard periods. PNU-7654 inhibits the Wnt signaling by preventing the interaction of Tcf and beta-catenin. PNU-74654 compound (Sigma Aldrich) was resuspended in Dimethyl Sulfoxide (DMSO; Sigma Aldrich) at stock concentrations of 31.2 mM, and cells were treated with vehicle (0.1%-0.4%) DMSO as a control. PNU-4654 was diluted 100X in a growth medium for working solutions and then diluted to 10 µM, 50 µM, and 100µM. Total RNA from NCI-H295 cells was isolated using TRIzol^®^ according to the manufacturer’s protocol (Life Technologies). RNA extraction and cDNA synthesis were performed as described above. For qPCR, TaqMan^®^ assay *NANOG* (Hs04260366_g1) was used according to the manufacturer’s recommendation using cDNA (diluted 1:5) as a template. TaqMan^®^ assay for NCI-H295 cells: Relative expression values were determined by the 2-ΔΔCt method using *GUSB* (ID: 4326320E) as endogenous control.

Our primary objective in this study was to explore the functional relevance of *NANOG* expression and the Wnt/β-catenin pathway inhibition in the context of adrenocortical carcinoma. We have not described dose-response curves or time-course studies because we employed a targeted experimental design based on concentrations and NCI-H295 cell viability in different times after PNU-74654 reported in prior literature ^(43)^. This previous study from our group demonstrated that this treatment in both concentrations significantly decreased viability of adrenocortical tumor cells. NCI-H295 cells were treated with PNU-74654, a beta-catenin inhibitor, with a range of concentrations: 5, 10, 50, 100 and 200 µM, and the effects were measured at different time points: after 24, 48, 72, and 96 hours. Higher concentrations (100 and 200 µM) caused a significant decrease in viability by 24–48 h. For lower concentrations, a reduction in viability became evident at later times (72–96 h). At 48 h, there was a clear dose-dependent increase in early and late apoptosis and reduced nuclear and cytoplasmic β-catenin accumulation versus vehicle. The highest concentrations used in these previous study (100–200 µM) could be questioned for cytotoxicity, but these doses did not affect Y1 mouse adrenal and HeLa non-adrenal cell lines under similar conditions, supporting specificity in blocking Wnt/β-catenin signaling.

### Statistical analysis

The Mann-Whitney test was used to compare mRNA expression levels between ACT samples and controls. The Kruskal-Wallis test was used for comparisons between mRNA expression and different tumor stages. Spearman’s test was used to analyze the correlations between the expression of evaluated genes. Log-rank test and Kaplan-Meier plot were used to analyze the association between gene expression and overall survival. In the analysis of the association between survival outcomes with mRNA expression levels, the ACT samples were categorized as increased (hyper-expressed) or decreased (hypo-expressed), based on whether it is higher or lower than the median gene expression 2-ΔΔCt of the control samples, and Kaplan-Meier curves and Log-rank analysis were performed. One-way ANOVA followed by Holm-Sidak’s multiple comparisons post-test was used to evaluate the differences between before and after treatment with all concentrations of PNU-74654. Non-continuous variables were expressed in the plots as median and interquartile range, and continuous variables were expressed as median and standard error (SEM). The significant limit for all the tests was established at 95% (p-value ≤ 0.05). Statistical analysis was performed using GraphPad Prism 6 (GraphPad, San Diego, CA).

## RESULTS

### NANOG, OCT4, SOX2 And STAT3 protein expression during the adrenal development

**Figure 2.**
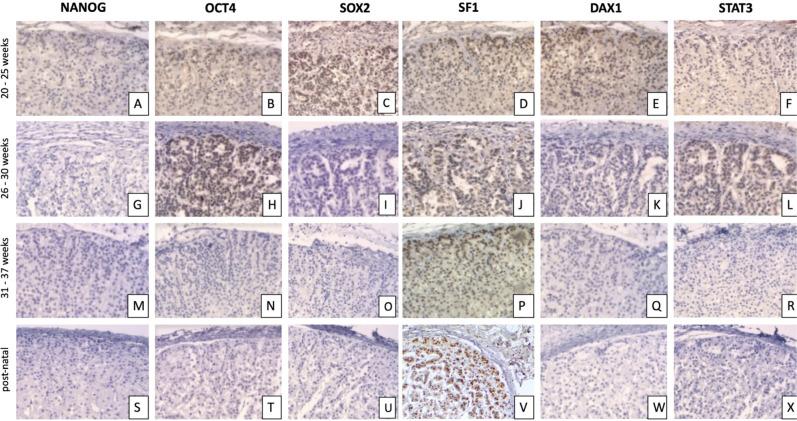
Immunohistochemistry (IHC) of NANOG, OCT4, SOX2, SF1, DAX1 and STAT3 protein expression during the adrenal development. IHC expression of NANOG at 20-25 weeks (**A**), 26-30 weeks (**G**), 31-37 weeks (**M**) of gestational period and postnatal (**S**). IHC expression of OCT4 at 20-25 weeks (**B**), 26-30 weeks (**H**), 31-37 weeks (**N**) of gestational period and postnatal (**T**). IHQ expression of SOX2 at 20-25 weeks (**C**), 26-30 weeks (**I**), 31-37 weeks (**O**) of gestational period and postnatal (**U**). IHQ expression of SF1 at 20-25 weeks (**D**), 26-30 weeks (**J**), 31-37 weeks (**P**) of gestational period and postnatal (**V**). IHQ expression of DAX1 at 20-25 weeks (**E**), 26-30 weeks (**K**), 31-37 weeks (**Q**) of gestational period and postnatal (**W**). IHQ expression of STAT3 at 20-25 weeks (**F**), 26-30 weeks (**L**), 31-37 weeks (**R**) of gestational period and postnatal (**X**).

We showed that all of the studied stem cell maintenance proteins (NANOG, OCT4, SOX2, and STAT3) were expressed between 20 and 25 weeks of gestational period (**Figures 2A, 2B, 2C and 2F**). After that, only OCT4 and STAT3 kept staining between 26 and 30 weeks (**Figures 2H and 2L**). However, none of these proteins were detected after 31 weeks of gestation and in the postnatal adrenal cortices **(Figures 2M, 2N, 2O and 2R**).

At 20-25 weeks of gestational age, the expression of the four proteins was located at the same adrenal layers but with different intensities. SOX2 stained strongly through the capsule and cortical regions (**Figure 2C**); OCT4 staining was stronger at the capsular region when compared with the rest of the cortex (**Figure 2B**); STAT3 staining was also observed in the capsule and cortex but in moderate intensity (**Figure 2F**); NANOG was expressed only in the capsular region and with weak intensity (**Figure 2A**). Between 26 and 30 weeks of gestational age, OCT4 and STAT3 kept the same pattern of expression as those between 20 and 25 weeks (**Figure 2H and 2L**), while NANOG and SOX2 were no longer expressed (**Figures 2G and 2I**). From 31 weeks till the end of gestation and in the postnatal life, OCT4 and STAT3 were also no longer expressed (**Figures 2N and 2O**).

### Clinical and pathological results

#### Pediatric patients

We evaluated 96 children (81% female) with a median age at the diagnosis of 1.6 years (0.4-15.6 years). Seventy-six patients (79%) were diagnosed in the first five years of life, including 19 (20%) diagnosed in the first year. Virilization due to androgen excess was the most common clinical finding in this group, and it was present in 92 patients (96%), including 51 patients with androgen-secreting tumors and 41 patients with both cortisol and androgen-secreting tumors (mixed tumors). Only four patients presented with non-secreting adrenal tumors, which were incidentally discovered (**Table 1**).

The germline p.R337H *P53* mutation was detected in 82 out of 96 pediatric patients (85%), and somatic exon 3 *CTNNB1* mutations were found in 10 out of 70 pediatric samples analyzed (14%).

Complete clinical and pathological features, including sex, age, clinical presentation, tumor stage, treatments, and death as an outcome, are described in **Table 1**. Part of these clinical data had been previously reported ^(1,4)^.

#### Adult patients

We evaluated 18 adults (89% female) with a median age at the diagnosis of 42.5 years (21-66 years). Cortisol-secreting tumors were found in 14 patients (77%), 50% of them being mixed cortisol and androgen-secreting tumors. Two patients presented with androgen-secreting tumors, and the other two patients had non-secreting tumors. According to the Weiss score tumor system, 44% of these tumors were carcinomas. The germline p.R337H *P53* mutation was detected in 4 patients (22%), and somatic *CTNNB1* mutations were detected in 7 patients (39%).

Clinical and pathological features, including sex, age, clinical presentation, tumor stage, treatment, and death, are described in **Table 2**.

### The stem cell marker proteins expression in ACT

#### pACT

**Figure 3.**
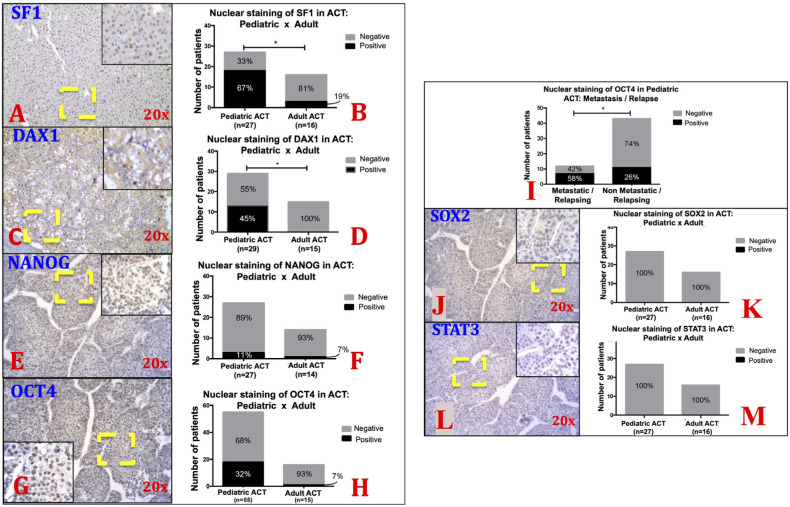
Immunohistochemistry in ACT: Pediatric vs Adult patients. (**A**) Positive nuclear staining for SF1. (**B**) Graphic depiction of the percentage of pediatric and adult ACT patients with positive nuclear staining for SF1 (*p<0.05). (**C**) Positive nuclear staining for DAX1. (**D**) Graphic depiction of the percentage of pediatric and adult ACT patients with positive nuclear staining for DAX1 (*p<0.05). (**E**) Positive nuclear staining for NANOG. (**F**) Graphic depiction of the percentage of pediatric and adult ACT patients with positive nuclear staining for NANOG. (**G**) Positive nuclear staining for OCT4. (**H**) Graphic depiction of the percentage of pediatric and adult ACT patients with positive nuclear staining for OCT4. (**I**) Percentage of OCT4 positive samples in metastatic/relapsing versus non-metastatic/non-relapsing ACTs (*p<0.05). (**J**) Nuclear staining for SOX2. (**K**) Graphic depiction of the percentage of pediatric and adult ACT patients with positive nuclear staining for SOX2. (**L**) Nuclear staining for STAT3. (**M**) Graphic depiction of the percentage of pediatric and adult ACT patients with positive nuclear staining for STAT3.

IHC for NANOG, OCT4, SOX2, and STAT3 was evaluated in a subset of pACT. We also evaluated a second tier of pACT samples to confirm the findings on OCT4 in the first cohort as described previously in the Methods section. Positive OCT4 nuclear staining was found in 18 out of 55 samples (32%) (**Figure 3H**). Interestingly, positive OCT4 was positively associated with metastatic pACT (odds ratio, 2.28; 95% confidence interval [CI]: 1.13 to 4.59; Fisher’s test: p-value < 0.05 – **Figure 3I**). Only three out of 27 samples (11%) presented with positive nuclear staining of NANOG (**Figure 3F**). All of the samples were negative for STAT3 and SOX2 staining (**Figures 3J and 3L**).

#### Adult ACT

IHC for NANOG, OCT4, SOX2, and STAT3 was evaluated in a subset of adult ACT (10 ACA, 6 ACC) samples (**Figures 3F and 3H, Figures 3K and 3M**). As in the pediatric samples, all adult samples were negative for STAT3 and SOX2 staining (**Figures 3K and 3M**). Only 1 out of 16 (7%) samples presented with positive nuclear staining of NANOG (**Figure 3F**). Positive OCT4 nuclear staining was also found in 1 sample (distinct from the one staining for NANOG) (**Figure 3H**).

### The Stem Cell Marker mRNA Expression

#### pACT

**Figure 4.**
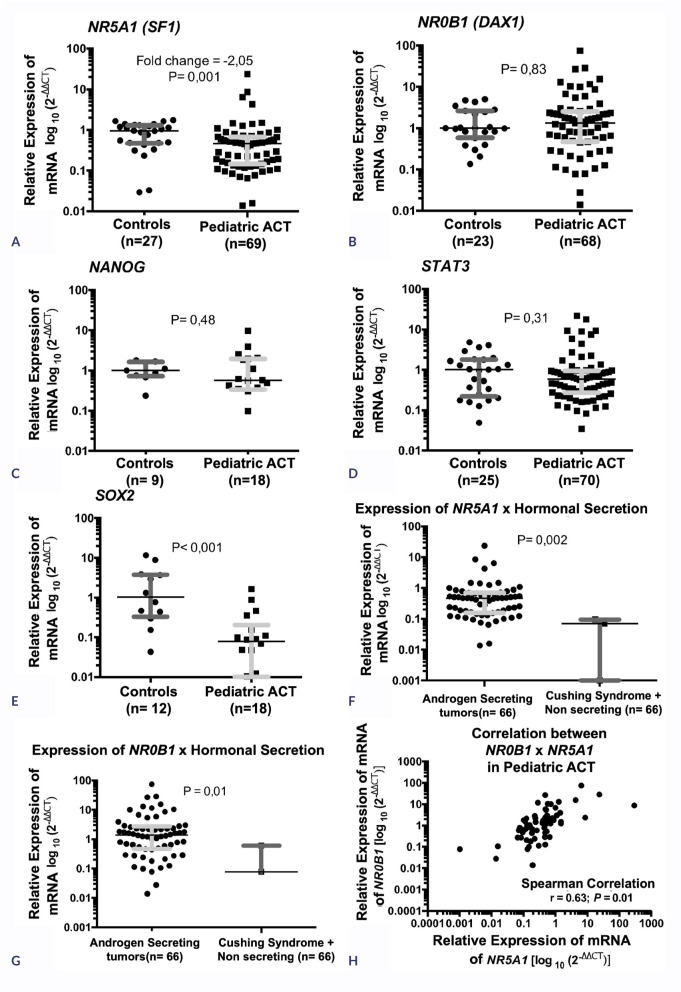
Gene expression in pACT. (**A**) *SF1* mRNA expression in control tissues and pACT tissues (p=0.001). (**B**) *DAX1* mRNA expression in control tissues and pACT tissues (p=0.83). (**C**) *NANOG* mRNA expression in control tissues and pACT tissues (p=0.48). (**D**) *STAT3* mRNA expression in control tissues and pACT tissues (p=0.31). (**E**) *SOX2* mRNA expression in control tissues and pACT tissues (p=0.001). (**F**) *SF1* mRNA expression in patients with androgen-secreting tumors and in patients with Cushing syndrome or non-secreting tumors. (**G**) *DAX1* mRNA expression in patients with androgen-secreting tumors and in patients with Cushing syndrome or non-secreting tumors. (**H**) Correlation between *DAX1* and *SF1* expression in pACT (r=0.63 and p=0.01).

Concerning the mRNA expression of stem cell maintenance transcription factors, pediatric patients presented with no difference in *STAT3* and *NANOG* expression between pACT and controls (p-value=0.31 and p-value=0.48, respectively – **Figures 4D and 4C**). pACT presented with lower *SOX2* mRNA expression when compared with controls (**Figure 4E** – p-value < 0.01). The mRNA expression of *OCT4* could not be analyzed because the cycle threshold (CT) of almost all the samples was undetermined.

#### Adult ACT

**Figure 5.**
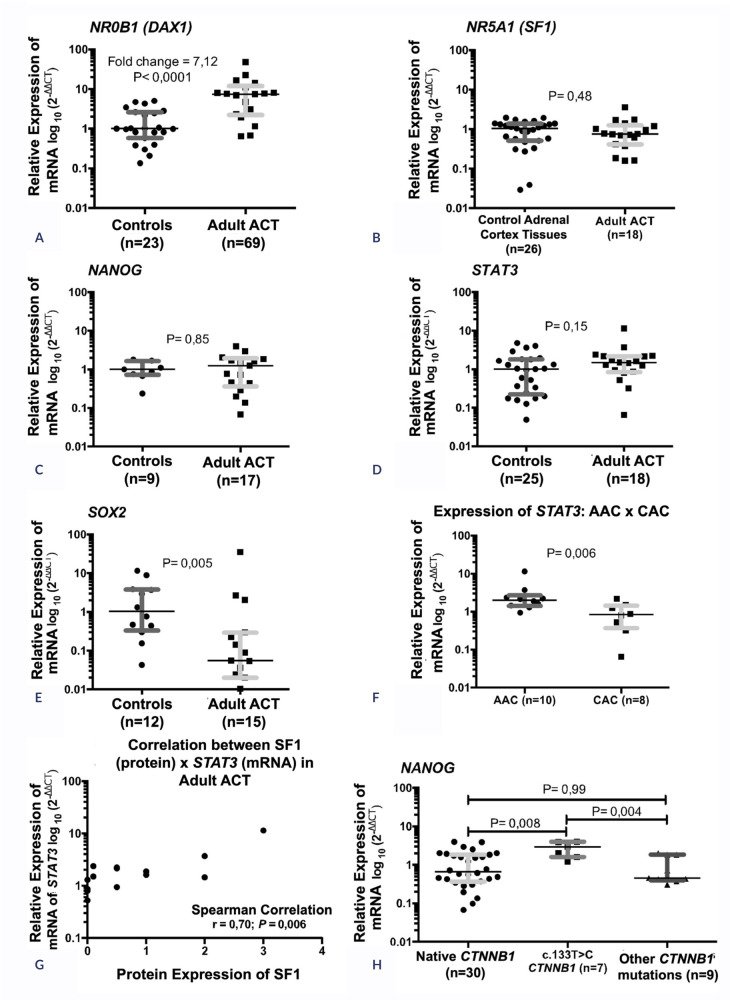
Gene expression in Adult ACT. (**A**) *DAX* mRNA expression in control tissues and adult ACT tissues (p=0.0001). (**B**) *SF1* mRNA expression in control tissues and adult ACT tissues (p=0.48). (**C**) *NANOG* mRNA expression in control tissues and adult ACT tissues (p=0.85). (**D**) *STAT3* mRNA expression in control tissues and adult ACT tissues (p=0.15). (**E**) *SOX2* mRNA expression in control tissues and adult ACT tissues (p=0.005). (**F**) *STAT3* mRNA expression in adenomas and in carcinomas. (**G**) Correlation between *STAT3* and *SF1* expression in pACT (r= 0.7 and p=0.006) (**H**) *NANOG* expression in patients with native *CTNNB1*, in patients with c.133T>C *CTNNB1* mutation, and inpatients with other *CTNNB1* mutations.

As in the pediatric group, adult ACTs presented with lower mRNA expression levels of *SOX2* (p-value < 0.01; **Figure 5E**) when compared to normal adrenal cortices. *STAT3* and *NANOG* expression did not differ (p-value = 0.15 and p-value = 0.85, respectively; **Figures 5D and 5C**) between adult ACT and control adrenal cortices. Curiously, cortisol-secreting tumors showed higher *STAT3* mRNA expression levels than other secreting tumors (p-value = 0.01). Among the adult ACT group, *STAT3* mRNA expression levels were higher in ACAs than in ACCs (p-value < 0.01; **Figure 5F**). However, neither the ACA nor the ACC groups had *STAT3* mRNA expression levels distinguished from control adult adrenal cortices (p-value = 0.13 and p-value = 0.77, respectively). Furthermore, survival was not different regarding *STAT3* mRNA expression levels (log-rank test, p-value = 0.87).

mRNA expression levels of *NANOG* were higher among patients carrying the p.S45P *CTNNB1* mutation (p-value < 0.01), in both adults and children (**Figure 5H**). No differential mRNA expression was observed in association with p.R337H *P53*. No association was observed among patterns of mRNA expression of any gene in this study, tumor stage, or overall survival in the adult or pACT groups.

#### In vitro data NANOG expression pattern in NCI-H295 cells

**Figure 6.**
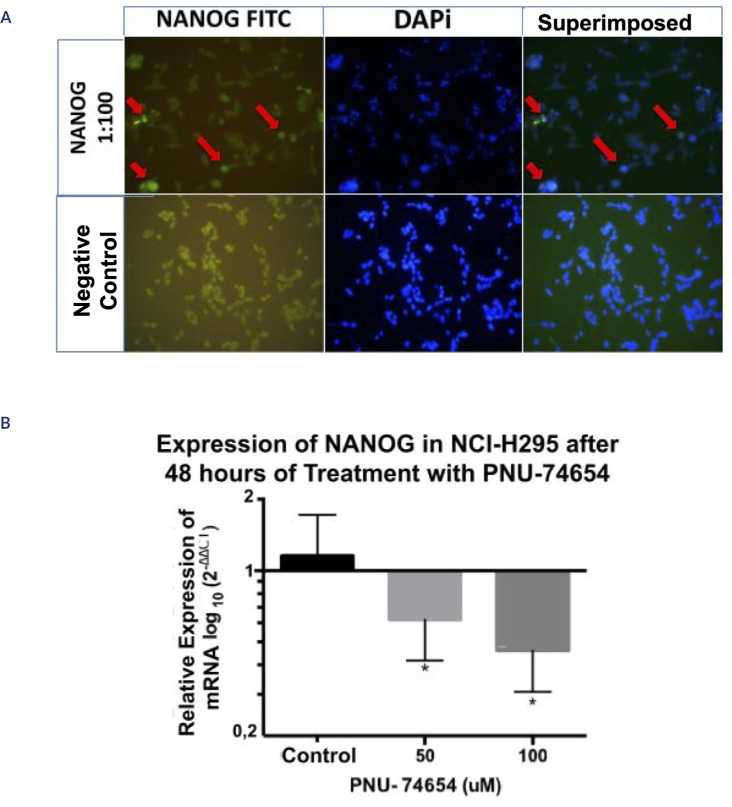
*NANOG* mRNA expression. (**A**) *NANOG* mRNA expression after inhibiting the Wnt/β-catenin signaling in NCI-H295 cells after 48hs treatment with PNU-74654 at 50 and 100 µM. (**B**) ICF shows NANOG staining in some NCI-H295 cells under basal conditions..

Considering the finding of higher *NANOG* mRNA expression in p.S45P *CTNNB1* mutated tumors, we performed in vitro tests using the NCI-H295 lineage, which carries this mutation, to evaluate where and if NANOG protein was expressed and its cellular location. On immunofluorescence, a bright green light was observed in the nucleus of some NCI-H295 cells (**Figure 6**), confirming that this protein is expressed in this lineage. As it was expressed in only a few cells, it seems that these may constitute a subgroup of cells with different characteristics.

#### Effect of the Wnt/beta-catenin signaling inhibition by PNU-7654 on NANOG mRNA expression levels in H295 cells

*NANOG* mRNA expression in NCI-H295 cells was detected under basal conditions. After 48h under PNU-74654 treatment at 50 and 100µM, a significant decrease in *NANOG* mRNA expression levels was observed (25% and 46% reduction, respectively; ANOVA: p-value < 0.01; **Figure 6**).

## DISCUSSION

The expression pattern of the stem cell maintenance genes NANOG, OCT4, SOX2, and STAT3 during the development of healthy human adrenal cortex in different stages of development or in ACT has not been further explored to date. In the present study, we analyzed the expression pattern of these stem cell maintenance genes in control adrenal cortices from the second half of gestation, the postnatal period, and in ACT from children and adults. Furthermore, we also performed an in vitro experiment to demonstrate that the Wnt/β-catenin pathway signaling regulates the *NANOG* expression in p.S45P β-catenin mutated ACT.

We observed that the human adrenal cortex cells still maintain the expression of NANOG, OCT4, and STAT3 after steroidogenesis begins, which occurs around week 7 of gestation in humans. It is different from what is observed in murine embryonic stem cells, in which the expression of these proteins ceases when adrenal steroidogenesis starts ^(23,44,45)^. These discrepancies likely reflect fundamental species differences in adrenal development, which were also previously described in mouse and primate gene-expression studies throughout embryological development ^(46,47)^. Therefore, while murine models provide valuable mechanistic insights, direct extrapolation to human development must be made with caution. In this context, our findings suggest that the human adrenal cortex may retain stemness-related transcriptional programs longer than the murine cortex. Our data also implies that the adult stem cell niche does not need *NANOG*, *OCT4*, *SOX2*, and *STAT3* activity to maintain itself, as the expression of these proteins was not observed in the postnatal adrenal cortices.

It is well known that almost all tissues possess adult stem cell niches, which serve to replace worn-out tissues ^(48)^. The adult stem cell niche in the adrenal cortex is located within and/or underneath the capsular region. A more intense expression of OCT4, SOX2, and STAT3 in the subcapsular region of the adrenal cortex between 20 and 31 weeks of gestation suggests that these markers may contribute to the establishment of the adult stem cell niche but not maintain it.

Increased OCT4 expression was found in one-third of the pACTs and was associated with metastatic disease. However, this finding was not observed in adult ACT. Previous data in the literature showed increased OCT4 expression associated with worse prognosis and chemotherapy resistance in several types of cancer. This data is consistent with our findings in metastatic tumors ^(49)^.

Both pediatric and adult ACTs presented with lower levels of SOX2 expression when compared to control tissues. Therefore, this marker might not be associated with the pathogenesis of adrenocortical tumors as it is in other types of cancer, such as breast and colorectal cancer ^(50,51)^.

STAT3 expression was higher in cortisol-secreting ACAs compared to other tumors, but no difference was observed compared to control tissues. STAT3 is crucial to the control of the anti-tumor immune response, and its hyperactivation is found in several types of cancer and is associated with a worse prognosis ^(52)^. Whether or not STAT3 has a role in the pathogenesis of cortisol-secreting pACT warrants further investigation.

mRNA expression levels of *NANOG* were higher among patients carrying the p.S45P *CTNNB1* mutation in adults and children, as confirmed in in vitro experiments. *NANOG* mRNA expression was decreased by inhibition of Wnt/beta-catenin signaling. Data in the literature have shown that *NANOG* can induce stemness, self-renewal, and invasive and metastatic behavior in some types of cancers through JAK/STAT and Wnt/beta-catenin ^(53)^, which is consistent with our findings in pACT and adult ACT. Wnt/β-catenin signaling has been shown to regulate *NANOG* expression via β-catenin/TCF complexes directly, and through interaction with OCT4, contributing to the maintenance of stemness and tumor-initiating capacity ^(54)^. Takao and cols. demonstrated that β-catenin up-regulates Nanog expression by directly interacting with Oct-3/4 in embryonic stem cells ^(54)^. In our study, we have observed a dose-dependent decrease in *NANOG* mRNA expression in the NCI-H295 cell line 48 hours after blocking the Wnt/β-catenin pathway with PNU 74654. Similar findings have been reported previously in lung cell lines ^(55)^ and small-cell lung cancer ^(56)^. In the latter, coexpression of *OCT4* and *NANOG* increased the self-renewal potential of cancer cells, increased drug resistance, and tumorigenic capacity through Wnt/β-catenin signaling activation. Our findings indicate an association between* NANOG* and *OCT4* expression and the activation of the Wnt/β-catenin pathway, which is linked with the pathogenesis of adrenocortical tumors.

This study has some limitations that should be acknowledged. First, because ACTs are rare, the adult cohort was necessarily small and based on a convenience sample, which limits statistical power and reduces the ability to detect subtle differences between groups. Second, acquiring control pediatric and adult tissues is challenging. We only obtained fetal tissue after 20 weeks of gestational age, and earlier gestational periods were not analyzed, and conclusions should not be drawn concerning early developmental periods. We had a heterogeneous control group that can influence the results. However, we applied exclusion criteria to minimize bias. Third, although *GUSB* was selected as the endogenous control for qPCR based on prior reports of its stability in similar tissues, we did not perform a formal reference gene stability assessment across all sample types, which might affect normalization accuracy. Fourth, we did not conduct an inter-observer reliability analysis for the IHC evaluations. Finally, the descriptive comparison of fetal and postnatal adrenal cortex IHC did not include statistical testing, limiting the strength of conclusions regarding developmental expression patterns. Despite these limitations, our study provides original and valuable data on a rare disease and suggests that stem cell–associated markers may contribute to the pathogenesis and prognosis of ACTs.

In conclusion, we found increased expression of OCT4, SOX2, and STAT3 in the subcapsular region of the adrenal cortex between 20 and 31 weeks of gestation, suggesting that these markers may contribute to the establishment of the adult stem cells niche but appear not to be essential to maintain it. OCT4 expression was increased in pACTs and was associated with a worse prognosis. Expression of *NANOG* was higher in both adult and pediatric patients with ACTs carrying p.S45P *CTNNB1* mutation, and its expression was decreased after Wnt/β-catenin signaling inhibition in an adrenocortical cell line harboring this mutation. *STAT3* expression was increased in cortisol-secreting ACTs, but whether or not it plays a role in pathogenesis or prognosis is unclear since there was no difference in gene expression when compared with controls. *SOX2* seems to not play a role in ACT pathogenesis.

## Data Availability

the data sets analysed in this study are included in the manuscript.

## SUPPLEMENTARY MATERIALS

**Supplementary table 1.** Characteristics of fetal and post-natal control samples

Sex	Gestational Age	Chronological Age	Cause of Death
M	22 weeks 5 days	-	Chorioamnionitis
F	22 weeks	-	Prematurity
M	22weeks 2 days	-	Prematurity/anoxia
F	25 weeks	-	Hyaline membrane syndrome
M	21 weeks	-	Anoxia
F	25 weeks	-	Anoxia/pre-eclampsia
M	22 weeks	-	Anoxia/chorioamnionitis
M	22 weeks	-	Anoxia
M	24 weeks	-	Prematurity/patent foramen ovale
M	21 weeks 4 days	-	Anoxia
M	21 weeks 4 days	-	Anoxia
M	21 weeks	-	Anoxia
F	24 weeks 4 days	-	Fetal distress
M	21 weeks 2 days	-	Prematurity
M	29 weeks	-	Diaphragmatic hernia
F	28 weeks 1 day	-	Pneumonia/anoxia
F	30 weeks 1 day	-	Anoxia
M	27 weeks	-	Anoxia/twin pregnancy
F	29 weeks	-	
F	26 weeks	-	Prematurity/amniorrhexis
F	26 weeks 1 day	-	Prematurity
M	29 weeks 6 days	-	Shock/pneumonia
F	35 weeks	-	Anoxia
M	35 weeks	-	Premature placental abruption
M	35 weeks 1 day	-	Sepsis/amniorrhexis
M	37 weeks	-	Diaphragmatic hernia/pulmonary hypoplasia
M	36 weeks 3 days	-	Anoxia
F	37 weeks	-	Premature placental abruption
F	37 weeks	-	Anoxia/pneumonia
M	34 weeks	-	Anoxia
F	-	4 days	Cardiopathy/anoxia
F	-	57 days	Cardiopathy
M	-	38 days	Cardiopathy
M	-	1 year	Septic shock
F	-	5 months	Sepsis
M	-	3.5 years	Encephalitis
M	-	3 months	Septic shock
M	-	5 months	Cardiopathy/shock
M	-	4 months	Post-operative following cardiac surgery
F	-	11 days	Cardiopathy/meningitis
M	-	2 years	Post-operative following cardiac surgery
M	-	8 days	Cardiac insuficiency/cardiopathy
F	-	1 month	Jejunual atresia/pneumonia
F	-	10 years	Post-operative following cardiac surgery

F: female; M: male.

**Supplementary table 2.** Characteristics of adult and pediatric control samples

Sex	Gestational Age	Chronological Age
F	51	Pulmonary embolism
M	41	Accute pancreatitis
M	54	Accute myocardial infarction
M	49	Acute pulmonary edema
M	48	Acute pulmonary edema
M	54	Accute myocardial infarction
M	44	Accute pancreatitis
M	36	Acute pulmonary edema
F	48	Pneumonia
M	45	Sudden Death
ND		
ND		
ND		
ND		
ND	75	Cardiac arrest
ND	0.01	
ND	0.04	
F	0.1	Wilms’ tumor surgical procedure
F	2.0	Wilms’ tumor surgical procedure
F	3.6	Wilms’ tumor surgical procedure
F	0.4	Wilms’ tumor surgical procedure
M	5.3	Wilms’ tumor surgical procedure
F	2.3	Wilms’ tumor surgical procedure
F	4.2	Wilms’ tumor surgical procedure
M	2.2	Wilms’ tumor surgical procedure
F	4.7	Wilms’ tumor surgical procedure

F: female; M: male; ND: no data.
